# The Impact of Positive Peritoneal Cytology on the Survival Rates of Early-Stage-Disease Endometrial Cancer Patients: Systematic Review and Meta-Analysis

**DOI:** 10.3390/medicina60050733

**Published:** 2024-04-28

**Authors:** Vasilios Pergialiotis, Michail Panagiotopoulos, Antonios Koutras, Andreas Daras, Thomas Ntounis, Michalis Liontos, Georgios Daskalakis, Nikolaos Thomakos

**Affiliations:** 1 First Department of Obstetrics and Gynecology, Division of Gynecologic Oncology, “Alexandra” General Hospital, National and Kapodistrian University of Athens, 11528 Athens, Greeceadrsdrs@gmail.com (A.D.); thomasntounis@gmail.com (T.N.); gdaskalakis@yahoo.com (G.D.); thomakir@hotmail.com (N.T.); 2 Department of Clinical Therapeutics, Alexandra General Hospital, School of Medicine, National and Kapodistrian University of Athens, 11528 Athens, Greece; mliontos@gmail.com

**Keywords:** endometrial cancer, peritoneal cytology, overall survival, recurrence-free survival, meta-analysis

## Abstract

*Background and Objectives:* The impact of positive peritoneal cytology has been a matter of controversy in early-stage endometrial cancer for several years. The latest staging systems do not take into consideration its presence; however, emerging evidence about its potential harmful effect on patient survival outcomes suggests otherwise. In the present systematic review and meta-analysis, we sought to accumulate current evidence. *Materials and Methods:* Medline, Scopus, the Cochrane Central Register of Controlled Trials CENTRAL, Google Scholar and Clinicaltrials.gov databases were searched for relevant articles. Effect sizes were calculated in Rstudio using the meta function. A sensitivity analysis was carried out to evaluate the possibility of small-study effects and p-hacking. Trial sequential analysis was used to evaluate the adequacy of the sample size. The methodological quality of the included studies was assessed using the Newcastle–Ottawa scale. *Results:* Fifteen articles were finally included in the present systematic review that involved 19,255 women with early-stage endometrial cancer. The Newcastle–Ottawa scale indicated that the majority of included studies had a moderate risk of bias in their selection of participants, a moderate risk of bias in terms of the comparability of groups (positive peritoneal cytology vs. negative peritoneal cytology) and a low risk of bias concerning the assessment of the outcome. The results of the meta-analysis indicated that women with early-stage endometrial cancer and positive peritoneal cytology had significantly lower 5-year recurrence-free survival (RFS) (hazards ratio (HR) 0.26, 95% CI 0.09, 0.71). As a result of the decreased recurrence-free survival, patients with positive peritoneal cytology also exhibited reduced 5-year overall survival outcomes (HR 0.50, 95% CI 0.27, 0.92). The overall survival of the included patients was considerably higher among those that did not have positive peritoneal cytology (HR 12.76, 95% CI 2.78, 58.51). *Conclusions:* Positive peritoneal cytology seems to be a negative prognostic indicator of survival outcomes of patients with endometrial cancer. Considering the absence of data related to the molecular profile of patients, further research is needed to evaluate if this factor should be reinstituted in future staging systems.

## 1. Introduction

Endometrial cancer is the most common gynecological cancer, with a global prevalence that surpassed 400,000 new cases globally in 2020, a number that increased by 0.69% compared to that reported in 1990 [1]. The American Cancer Society reported that the incidence of cancer of the uterine corpus reached 27.7 cases per 100.000 people, and researchers estimated that approximately 66,200 women would be diagnosed with endometrial cancer in 2023 in the United States [2]. Wide variations in its prevalence are observed in a worldwide setting that seem to be influenced by socioeconomic factors, with a global age-standardized rate (ASR) of 8.7/100.000 that easily exceeds the ASR limit of 20/100.000 in countries with an increased-prevalence overweight population [3]. The majority of endometrial cancer cases are diagnosed in the early stage, as the primary symptom, namely postmenopausal bleeding, is encountered early in the course of the disease. In these cases, the management is largely influenced in our era by actual histological features as well as by novel molecular indices that help predict the response to chemotherapy and the actual progression-free (PFS) and overall survival (OS) of patients.

Positive peritoneal washings have been previously linked to the possibility of microscopic peritoneal metastases [4], and considering the impact of positive peritoneal cytology on the survival rates of gynecological cancer patients [5], the presence of this feature has been adopted as a predictive factor of survival in endometrial cancer as well by the International Federation of Gynecology and Obstetrics (FIGO). In 2009, peritoneal cytology was removed from the staging system [6]. Despite the fact that the authors did not justify this decision, it must have been based on articles that contested its correlation with the survival of patients with early-stage disease [7,8]. Since then, several articles have been published; however, to date, accumulated evidence concerning the importance of positive peritoneal cytology in early-stage disease is still missing. The current FIGO classification system thoroughly assessed several factors that were not previously documented, including the presence of lymphovascular space involvement, mutation of the p53 gene and the presence of polymerase E (POLE) mutations [9]. Specifically, this classification suggests that patients with early-stage disease must be grouped into stage II when LVSI and/or aggressive histotypes are present, even in the absence of cervical stromal invasion, thus denoting the importance of detecting patients with several other cofactors that seem to influence the survival outcomes of patients with early-stage disease. Considering this information and taking in mind the conflicting evidence regarding the potential effect of positive peritoneal cytology in early-stage disease, in the present systematic review, we seek to investigate the correlation of positive peritoneal washings with other known factors that affect the survival of patients with disease that is otherwise confined to the uterine body and uterine cervix and to evaluate the impact of these findings on patients’ survival rates.

## 2. Materials and Methods

The present systematic review was registered in PROSPERO (International prospective register of systematic reviews) prior to its onset (Registration number: CRD42022308702) and is designed according to the Preferred Reporting Items for Systematic Reviews and Meta-Analyses (PRISMA) guidelines [10]. The review is based on aggregated data that have already been published in the international literature. Patient consent and institutional review board approval were, therefore, waived. 

### 2.1. Eligibility Criteria, Information Sources, and Search Strategy

The eligibility criteria for the inclusion of studies were predetermined. All studies that examined the impact of positive peritoneal cytology in patients with disease that was otherwise confined to the uterus (FIGO 2018 stages I and II) were considered eligible for inclusion. All histology groups were considered eligible for inclusion; however, a decision to proceed with subgroup analysis was taken if subgroup data for low-grade endometrioid tumors and other tumors were available. The molecular classification was not considered for the purposes of the present systematic review due to the limited number of articles that was expected to be retrieved. Case reports, experimental studies and conference proceedings were excluded from the present meta-analysis. 

**Figure 1:**
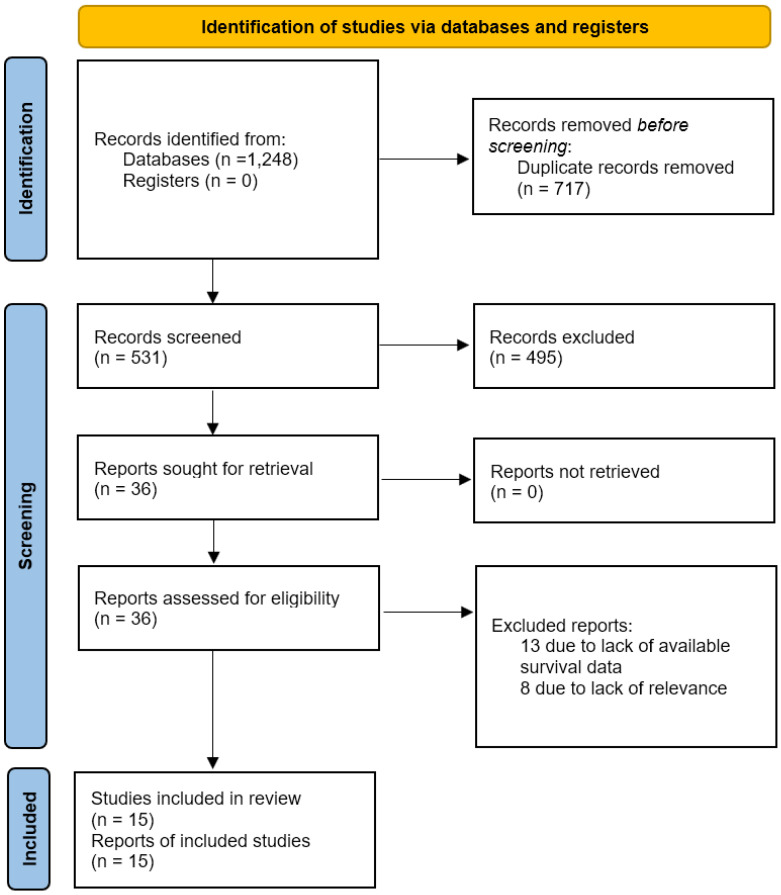
Search plot diagram.

We used the Medline (1966–2023), Scopus (2004–2023), Clinicaltrials.gov (2008–2023), Cochrane Central Register of Controlled Trials CENTRAL (1999–2023) and Google Scholar (2004–2023) databases in our primary search along with the reference lists of electronically retrieved full-text papers for articles published in the Latin alphabet, regardless of the actual language. A decision to translate languages other than English, French, German, Italian and Spanish with online translating tools was taken before the onset of the search. The date of our last search was set at 20 December 2023. Our search strategy included the text words “endometrial cancer; uterine cancer; cytology; peritoneal washings; survival” and is briefly presented in Figure 1.

### 2.2. Study Selection

The retrieved studies were selected in three consecutive stages. The first step involved the deduplication of the retrieved articles, which was followed by manual screening by two authors (MP and VP) of the titles and abstracts of all electronic articles that remained to evaluate their eligibility. Finally, studies that were considered potentially eligible were selected for inclusion following retrieval and review of the full text. Discrepancies that arose in this latter stage were resolved by consensus from all authors. 

### 2.3. Data Extraction

Outcome measures were predefined during the design of the present systematic review. Data extraction was performed using a modified data form that was based on Cochrane’s data collection form for intervention reviews for randomized controlled trials (RCTs) and non-RCTs [11]. The primary outcome of our study was the evaluation of differences in survival rates (overall survival (OS) and recurrence-free survival (RFS)) of endometrial cancer patients with early-stage disease with or without positive peritoneal cytology. Differences in crude time intervals until disease relapse and death were considered as secondary outcomes as well as differences in other histological parameters among patients with positive or negative peritoneal cytology, including histological subtype and the presence of lymphovascular space involvement.

### 2.4. Assessment of Risk of Bias

The methodological quality of the included observational studies was assessed by two authors (LV and VP) using the Newcastle–Ottawa scale (NOS) score that assesses the risk of bias in observational studies by evaluating the selection of the study groups (maximum rating: 4 points), the comparability of the groups (maximum rating: 2 points—1 for comparable histological subtypes and another 1 for the level of myometrial invasion and/or cervical involvement) and the outcome of interest (which was predefined as a minimum of 3 years of median follow-up (indicated either by the authors or by an interval ≥ 4 years between the last patient recruitment and publication of the article)) (maximum rating: 3 points) [12].

### 2.5. Data Synthesis

Statistical meta-analysis was performed with RStudio using the *meta* function (RStudio Team (2015); RStudio: Integrated Development for R; RStudio, Inc., Boston, MA; URL: http://www.rstudio.com/, accessed on 1 December 2023). Statistical heterogeneity was not considered during the evaluation of the appropriate model (fixed effects or random effects) of statistical analysis as the considerable methodological heterogeneity (Table 1) did not permit the assumption of comparable effect sizes among studies included in the meta-analysis [13]. Confidence intervals were set at 95%. We calculated pooled odds ratios (ORs), hazards ratios (HRs), and mean differences (MDs) of survival as well as pooled 95% confidence intervals (CIs) with the Hartung–Knapp–Sidik–Jonkman instead of the traditional Dersimonian–Laird random effects model analysis (REM). The decision to proceed with this type of analysis was taken after considering recent reports that support its superiority compared to the Dersimonian–Laird model when comparing studies of varying sample sizes and between-study heterogeneity [14]. Publication bias was initially designed to be assessed using inspection of retrieved funnel plots for outcomes that included more than 10 studies as well as with the Egger’s test, which represents a linear regression analysis that takes into account the intervention effect estimates and their standard errors, which are weighted by their inverse variance [15]. 

The potential presence of small-study effects was evaluated with Rücker’s Limit Meta-Analysis, and the possibility of p-hacking was evaluated with inspection of the results of the p-curve analysis.

#### 2.5.1. Prediction Intervals

Prediction intervals (PIs) were also calculated, using the *meta* function in RStudio, to evaluate the estimated effect that is expected to be seen by future studies in the field. The estimation of prediction intervals considers the inter-study variation in results and expresses the existing heterogeneity at the same scale as the examined outcome. 

#### 2.5.2. Trial Sequential Analysis

To evaluate the information size, we performed trial sequential analysis (TSA) in all meta-analysis that involved binary or continuous outcomes, which permits investigation of the type I error in the aggregated result of meta-analyses performed for primary outcomes that were predefined in the present meta-analysis. A minimum of 3 studies was considered as appropriate to perform the analysis. Repeated significance testing increases the risk of type I error in meta-analyses and TSA has the ability to re-adjust the desired significance level by using the O’Brien–Flemming a-spending function. Therefore, during TSA, sequential interim analyses are performed that permit investigation of the impact of each study in the overall findings of the meta-analysis. The risk for type I errors was set at 5% and for type II errors at 20%. Trial sequential analysis was not performed for pre-calculated effect size data, namely hazards ratios, provided in this meta-analysis, as, currently, there is no available algorithm for these types of data. The TSA was performed using the TSA v. 0.9.5.10 Beta software (http://www.ctu.dk/tsa/, accessed 20 December 2023).

## 3. Results

Thirty-six articles were identified from the literature search. Of those, 21 articles were excluded as they involved combined populations with early- and late-stage disease or did not report the outcomes of interest [29,30,31,32,33,34,35,36,37,38,39,40,41,42,43,44,45,46,47,48,49]. Fifteen articles were finally included in the present systematic review [7,16,17,18,19,20,21,22,23,24,25,26,27,28,35] that involved 19,255 women with early-stage endometrial cancer. Significant discrepancies were observed in the methodological characteristics of the included studies, which were anticipated due to the changes that were made in the FIGO classification of endometrial carcinoma and the treatment that was offered to these women over the span of 40 years that refers to the studies included (Table 1). The reporting of tumor characteristics and adjuvant treatment offered to the series of patients included in each study differed, with significant gaps observed in their majority (Table 2). The Newcastle–Ottawa scale indicated that the majority of included studies had a moderate risk of bias in their selection of participants, a moderate risk of bias in terms of the comparability of groups (positive peritoneal cytology vs. negative peritoneal cytology) and a low risk of bias concerning the assessment of the outcome (Table 3). 

**Figure 2:**
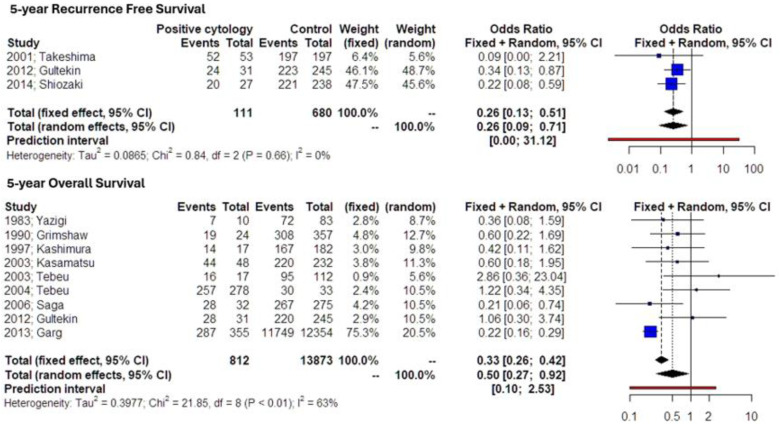
Hazards ratio of recurrence-free (upper forest plot) and overall survival (lower forest plot). Forest plot analysis: Vertical line = “no difference” point between the two groups. Blue squares = hazard ratios of included studies; horizontal black lines = 95% CI of included studies; diamond = pooled hazard ratios retrieved from the outcomes of the meta-analysis and 95% CIs for all studies; horizontal red line = prediction intervals. The weight of included studies is depicted for fixed- and random-effects models separately. Moderate statistical heterogeneity is observed for the overall survival outcome (I-square = 63%) and no statistical heterogeneity is noted for recurrence-free survival (I-square = 0%).

The results of the meta-analysis indicated that women with early-stage endometrial cancer and positive peritoneal cytology had significantly lower 5-year RFS (HR 0.26, 95% CI 0.09, 0.71, Figure 2). Prediction intervals indicated that the data were not sufficient to support this effect in future studies. The level of statistical heterogeneity was, however, particularly low (*I-square test* = 0%), which resulted in comparable summary effect estimates with the fixed-effects model (HR 0.26, 95% CI 0.13, 0.51). Further sensitivity analysis did not indicate potential outliers. P-curve analysis revealed that p-hacking could not be entirely ruled out, indicating the need for further evidence.

As a result of the decreased recurrence-free survival, patients with positive peritoneal cytology also exhibited reduced 5-year overall survival outcomes (HR 0.50, 95% CI 0.27, 0.92, Figure 2). The large prediction intervals resulted in an underpowered effect that was not statistically significant. This could be attributed to the large standard errors of individual studies and was confirmed by Rucker’s analysis, which indicated a particularly significant small-study effect (*p* < 0.001). Following adjustment, the summary estimate resulted in a significantly larger effect size with narrower prediction intervals (HR 0.21, 95% CI 0.15, 0.32) that was completely free of residual heterogeneity beyond small-study effects (*p* = 0.454). To further observe if this might be the result of potential outliers, we performed the relative analysis, which did not reveal any significant data. The p-curve analysis could not be performed due to the absence of available data. 

Lastly, the overall survival of included patients was considerably higher among those that did not have positive peritoneal cytology (HR 12.76, 95% CI 2.78, 58.51). Substantial statistical heterogeneity was noted (*I-square test* = 99%). Prediction intervals revealed that future studies might not prove this association; however, these were particularly wide, something that could be attributed to potential outliers. Following outlier analysis, one study was excluded from the random-effects model [10] and the result of the summary effect estimate remained significant, albeit somewhat reduced (HR 9.23, 95% CI 1.86, 45.85). This indicated the potential presence of small-study effects. After performing Rucker’s meta-analysis, a significant impact of the standard errors of individual studies was observed, and the adjusted effect estimate following correction for small-study effects resulted in particularly narrower confidence intervals (HR 15.44, 95% CI 12.36, 19.24) with absent residual heterogeneity (*p*-value = 0). P-curve analysis indicated the presence of evidential value and the absence of potential p-hacking.

## 4. Discussion

The findings of our study suggest that women with early-stage disease may have worse survival outcomes when peritoneal cytology is positive. The overall effect estimates that were provided for the 5-year recurrence-free and overall survival were particularly large, indicating that women with negative peritoneal cytology had an 80% reduction in recurrence rates and a 12-fold increase in their overall survival. Statistical heterogeneity was noted, which could be attributed to small-study effects, something that was anticipated due to the relatively small number of participants that were included in the studies that were retrieved. Of note, however, even after correction, the effect estimate remained particularly large and the p-curve analysis did not reveal evidence of p-hacking that would render our findings questionable. 

The removal of peritoneal cytology from the FIGO 2009 staging system of endometrial cancer was based on studies that revealed the absence of a potential correlation. However, the largest study published to date, which was based on data from the Surveillance, Epidemiology and End Results (SEER) database of the U.S. National Cancer Institute, revealed the presence of significant differences in recurrence-free survival [10]. This could explain the decreased overall survival that was observed by smaller case–control studies which had the inherent problem of selection bias that was noted in our systematic review [13,33,35].

Several studies were published indicating conflicting findings regarding the need for evaluation of peritoneal cytology in early-stage endometrial cancer. These data are supported by the largest study published to date that was based on 6313 endometrial cancer cases, of whom 384 patients had positive peritoneal cytology [49]. With a median overall survival of 44 months, the authors revealed that patients with positive peritoneal cytology (PC) were more likely to undergo laparoscopy (58.1% of cases) and radical hysterectomy (17.7% of cases). Furthermore, these patients were more frequently diagnosed with high-risk factors, such as high-grade tumors (25%) and a myometrial invasion depth greater than half (27.1% of cases), as well as advanced FIGO stage (III/IV) (41.5%). According to the results of multivariate Cox proportional hazards models, positive PC was found to be an independent risk factor for both post-operative survival (PFS) and overall survival (OS) (hazard ratio for PFS = 2.20, *p* < 0.001; hazard ratio for OS = 2.25, *p* < 0.001). Furthermore, in this study, the authors could provide a detailed account of the statistical significance of numerous risk factors and treatment outcomes in relation to the PC status. These included specific 5-year progression-free survival and overall survival rates, as well as hazard ratios for survival. There was also a direct connection between the presence of peritoneal cytology and the outcomes of survival and the treatment choices that were made. This connection was supported by extensive statistical evidence, which suggested that a positive PC significantly worsened the prognosis and ought to influence the decisions that were made regarding post-operative adjuvant therapy, particularly in intermediate and high–intermediate risk groups.

In another study conducted by Matsuo et al., the authors focused on treatment alternatives in connection to the status of peritoneal cytology and the impact that these modalities had on survival [41]. The shift in treatment practices over time, which included an increase in hormone therapy from 2.7% to 4.5% and a drop in systemic chemotherapy from 15.5% to 11.1%, was a reflection of the shifting therapeutic techniques. On the other hand, the survival analysis demonstrated that neither hormone therapy nor systemic chemotherapy significantly modified the 5-year survival results when compared to the situation in which no adjuvant therapy was administered. The relevance of this finding lied in the fact that it suggested that the predictive significance of peritoneal cytology may have not directly influenced the effectiveness of these treatments in terms of improving survival rates. Although it highlighted the predictive significance of peritoneal cytology in early-stage endometrial cancer, it also emphasized that better survival outcomes were associated with a negative cytology. Despite the fact that our study as well as the Matsuo et al. study approached the topic from different perspectives, both studies emphasized the significance of peritoneal cytology as a predictive factor in endometrial cancer. While, in our study, we stressed the impact of peritoneal cytology on survival outcomes and staging, Matsuo et al. presented a comprehensive statistical study that not only underlined the function that PC plays in prognosis but also outlined the ways in which it impacted different risk groups in a different way, with a particular emphasis on the 5-year survival rates. Taking all of these data into perspective, it was clear that the treatment planning and prognosis evaluation of endometrial cancer patients should take into account the patient’s PC status in a nuanced manner.

Another meta-analysis that was previously published proposed that positive peritoneal cytology could be a viable prognostic factor in endometrioid endometrial cancer due to the fact that it had a substantial connection with other prognostic factors and there was a considerable influence on survival. Both studies agreed on the adverse predictive importance of post-operative peritoneal cytology in endometrioid endometrial cancer, strengthening its link to poorer survival rates and increased occurrences of unfavorable prognostic markers such as grade 3 tumors and deep myometrial invasion [39]. However, in this study, the authors based the survival outcomes on a considerably smaller number of studies (two per evaluated outcome), therefore rendering the conduct of the present systematic review necessary. Specifically, Lee B et al.’s study concentrated more on determining the link between PPC and certain adverse prognostic variables (grade 3, profound myometrial invasion and lymphovascular space invasion) and indicated a requirement for additional assessment to elucidate its prognostic importance. In contrast, our study expanded the knowledge related to the 5-year progression-free and overall survival rates by focusing on these findings, therefore highlighting the substantial statistical proof that confirms the negative prognostic effect of positive peritoneal cytology on these crucial outcomes. Concerning survival outcomes, Lee et al. indicated that only the 5-year recurrence-free survival was considerably affected (HR 4.22 95% CI, 2.34, 7.61), whereas the importance of a positive peritoneal cytology finding did not significantly affect the 5-year overall survival (HR 2.88 95% CI, 1.24, 6.66). It should be noted, however, that survival analyses were performed using the fixed-effects Mantel–Haenszel model, a parameter that might have significantly skewed their findings due to the considerable heterogeneity that was evident in terms of population and tumor characteristics. This is supported by the fact that, in our study, we observed the presence of significant statistical heterogeneity, whereas in the previous meta-analysis, the authors did not report this important aspect of the statistical analysis.

Another large cohort study that was conducted by Seagle et al. based on data retrieved by the National Cancer Database also supported these findings, as the authors observed that the presence of positive peritoneal cytology significantly decreased the overall survival of patients with early-stage endometrial carcinoma (64.9 months vs. 60.6 months, *log-rank* < 0.001) [50]. The negative prognostic correlation of positive cytology remained consistently strong and unaffected by variations in sensitivity analyses, different models, changes in variables, or potential unmeasured confounding. The findings also remained consistent for the population with low-grade endometrioid cancer. However, the reported decrease in the overall survival outcomes was lower compared to that observed in our meta-analysis as well as the large cohort of the SEER (Surveillance, Epidemiology, and End Results Program) authors [23] (HR 1.85, 95% CI 1.54, 2.21).

### 4.1. Strengths and Limitations

Our study is based on a systematic review of the literature using a thorough search that included several databases. Strict criteria were used for the definition of early-stage disease, as from the initial search, it was observed that several studies reported results based on the clinical definition of early-stage disease, rather than relying on the actual pathology findings. These were excluded, as they reported outcomes that were also based on patients with advanced-stage disease (including those with peritoneal or lymphatic metastases). To evaluate the potential impact of differing techniques of patient management with the evolution of time, we performed outlier analysis to provide an accurate summary effect estimate following the exclusion of such studies. The provided outcomes confirmed the significance of our findings, therefore indicating that peritoneal cytology should be re-evaluated in patients with early-stage endometrial cancer. 

Nevertheless, the vast time span of the included studies renders the interpretation of our findings somewhat problematic due to the absence of data related to the use of adjuvant therapy. The omission of this important parameter in the included studies rendered the evaluation of the actual impact of different treatment plans on patients’ survival impossible and might explain the absence of a statistically significant effect in earlier studies, which could report outcomes based on a population that received adjuvant treatment in the presence of positive peritoneal cytology. 

### 4.2. Implications for Current Clinical Practice and Future Research

Considering these findings, we believe that the importance of positive peritoneal cytology should be thoroughly re-evaluated in cases with early-stage endometrial carcinoma as there is robust evidence that supports its negative prognostic impact. Ideally, future studies should focus on specific subtypes of endometrial cancer, including cases with low-grade and high-grade disease. Currently, with the evolution of molecular profiling in endometrial cancer, a novel staging system has been instituted that takes into account the classification provided by studies that performed TCGA (The Cancer Genome Atlas) analysis [9]. Stratification of women with early-stage disease is of particular importance, and to accomplish this, a series of histological and molecular factors are taken into account. From the molecular perspective, polymorphisms in the polymerase E (POLE) gene are considered less harmful, resulting in an ultramutated profile that has an indolent course, whereas mutations of the p53 gene are associated with aggressive neoplasms that usually progress early [51]. Tumors harboring mutations that result in microsatellite instability fall somewhat between these previous forms of tumors; however, they seem to respond well to program death ligand (PDL-1) antitumor therapy, even among cases with advanced-stage disease and disease relapse [52]. Considering this, it would be prudent to evaluate whether the presence of positive peritoneal cytology is associated with more aggressive molecular forms of endometrial cancer as well as to observe if its presence significantly affects the course of patients subgrouped according to the molecular profile of their tumor. 

Another interesting clinical aspect that deserves further investigation in the near future is the impact of post-hysteroscopy positive peritoneal cytology, as none of the studies included in the present systematic review reported this correlation. To date, our understanding of how hysteroscopy affects the diagnosis and staging of endometrial cancer has been significantly expanded by smaller studies that provide opposing results [53,54,55]. The rationale behind the hypothesis of a potential harmful effect of hysteroscopy on the survival outcomes of patients with endometrial cancer relies on the possibility of transtubal reflux, which has been observed particularly when 32% dextran is used as a distension medium [56]. This effect seems to be somewhat contained when normal saline is used [57,58], although some cells continue to pass through the tubal lumen. Taking into account the observed arbitrary effect of positive peritoneal cytology on the course of early-stage endometrial carcinoma, supported by the earlier studies included in the present meta-analysis, researchers frequently suggested in the past that hysteroscopy should not be considered a contraindication in the absence of metastatic disease [59]. Cytologically, there seems to be some evidence against the potential viability of extruded cells as well [35]; however, given the limited amount of data, the outdated techniques that were considered, and the emergence of novel evidence that supports the potential harmful effect of positive peritoneal cytology on the course of the disease, it seems evident that further research is needed to specifically target patients with stage I and/or II disease and substratify them by taking into consideration the molecular profile of the tumors, as the viability of cancer cells may differ among different subtypes of cells. 

## 5. Conclusions

Positive peritoneal cytology seems to directly impair the survival outcomes of patients with early-stage endometrial carcinoma. This information seems to be free from potential bias and does not rely on small-study effects, which could be the result of increased effect estimates retrieved from underpowered studies. However, significant heterogeneity is noted among the studies that were included in this meta-analysis, which is the result of the wide time span that was selected. Therefore, different treatment alternatives might be used among patients with positive peritoneal cytology, including expectant management and chemotherapy, with the latter being more prominent in earlier studies, when the disease was upstaged as stage IIIa(cyt). This is why prospective clinical trials are needed to indicate whether patients with positive peritoneal cytology should receive adjuvant treatment, and these should ideally focus on the molecular profile of tumors, which might significantly affect the course of the disease. 

## Figures and Tables

**Table 1:** Methodological characteristics of included studies.

Year	Author	Patient n	Years Screened	FIGO Staging	Inclusion Criteria/Treatment Characteristics
1983	Yazigi [16]	93	1958–1967	NR	Stage I endometrial cancer TAH-BSO
1990	Grimsaw [17]	381	NR	NR	NR
1997	Kashimura [18]	303	1978–1994	1988	Stage I–IV endometrial cancer; Stage I: TAH-BSO, BPLND and PALND; Stage II: RAH-BSO and BPLND and PALND; Stages III–IV: Palliative hysterectomy + radio + chemotherapy.
1997	Descamps [19]	201	1976–1992	1971	Stage I and II endometrial cancer; No other malignant disease prior to referral; No metastatic disease at presentation; Referral for primary treatment; Treated with RAH-BSO and BPLND.
2001	Obermair [20]	369	1995–1998	1988	Stage I endometrioid adenocarcinoma; No other malignant disease prior to referral; No metastatic disease at presentation; No serosal, vaginal or cervical involvement; No positive pelvic lymph nodes; Treated with TAH-BSO, BPLND and PALND; Follow up > 1 month.
2003	Κasamatsu [7]	280	1990–1998	1988	Stage I—IIIA endometrioid endometrial cancer; Treated with TAH-BSO + BPLND + PALND or TAH-BSO or RAH.
2006	Saga [21]	307	1998–2001	1988	Stage I and II endometrioid endometrial cancer; Stage I: TAH-BSO, BPLND and PALND; Stage II: RAH-BSO and BPLND and PALND.
2012	Gultekin [22]	351	1994–2009	1988 and 2009	Endometrial cancer; Treated with TAH-BSO, BPLND and PALND.
2013	Garg [23]	14,704	1988–2005	2009	Stage I and II endometrial cancer; No other malignant disease prior to referral; No metastatic disease at presentation; Treated with TAH-BSO, BPLND and PALND.
2001	Takeshima [8]	543	1980–1997	NR	Stage I–IV endometrioid endometrial cancer; Treated with TAH-BSO + BPLND+ PALND.
2003	Tebeu [24]	295	1980–1993	1988	Stage I–II endometrial cancer; Treated with TAH-BSO + BPLND+ PALND.
2004	Tebeu [25]	331	1980–1996	1988	Stage I–III endometrial cancer; Treated with TAH-BSO + BPLND+ PALND.
2014	Shiozaki [26]	265	1998–2010	2008	Stage I endometrial cancer; Treated with TAH-BSO + BPLND+ PALND.
2022	Siegenthaler [27]	124	NR	2009	Stage I–IV endometrial cancer; Treated with TAH-BSO + BPLND.
	Ueno [28]	908	1988–2012	2009	Stage I–IV endometrial cancer; Treated with TAH-BSO + BPLND.

Data from studies that included patients with early-stage and advanced-stage disease were considered only if subgroup analysis of survival outcomes was available from patients with early-stage disease. TAH-BSO: total abdominal hysterectomy and bilateral salpingo-oophorectomy, BPLND: bilateral pelvic lymph node dissection, PALND: para-aortic lymph node dissection, NR: not reported.

**Table 2:** Histological tumor characteristics and reporting of adjuvant treatment.

Year; Author	Histological Grade	Myometrial Invasion	Cervical Invasion	LVSI	Adjuvant Radiation	Adjuvant Chemotherapy
1990; Grimsaw [17]	NR	NR	NR	NR	NR	NR
1997; Kashimura [18]	NR	<50%: 20% vs. 80% >50%: 12% vs. 78% *	NR	NR	NR	NR
1997; Descamps [19]	NR	NR	NR	NR	NR	NR
2001; Obermair [20]	Gr1: 30.8% vs. 35.7% Gr2: 53.8% vs. 45.5% Gr3: 15.4% vs. 18.8%	None: 7.7% vs. 18% <50%: 84.6% vs. 56.7% >50%: 7.7% vs. 25.3%	NR	NR	46.2% vs. 28.4%	NR
2003; Κasamatsu [7]	Gr1: 81% vs. 63% Gr2: 17% vs. 24% Gr3: 2% vs. 13% *	None: 10% vs. 15% <1/3: 42% vs. 46% 1/3–2/3: 23% vs. 22% >2/3: 25% vs. 17% *	Absent: 70% vs. 85% Mucosal: 15% vs. 3% Stromal: 15% vs. 12% *	Negative: 71% vs. 74% Positive: 29% vs. 26%	NR	NR
2006; Saga [21]	Gr1: 59% vs. 67% Gr2 and 3: 41% vs. 43%	<50%: 72% vs. 72% >50%: 28% vs. 28%	Absent: 72% vs. 89% Present: 28% vs. 11%	Negative: 66% vs. 72% Positive: 38% vs. 28%	NR	NR
2012; Gultekin [22]	NR	NR	NR	NR	NR	NR
2013; Garg [23]	Gr1: 14.6% vs. 32.3% Gr2: 34.6% vs. 35% * Gr3: 40.2% vs. 23.8% Unknown: 10.5% vs. 8.8%	NR	NR	NR	No: 44.3% vs. 69.1% * Yes: 53.4% vs. 29.7% Unknown: 2.3 vs. 1.2%	NR
2001; Takeshima [8]	Gr1 and <50% myometrial invasion: 9.7% vs. 36.2% Gr2 or Gr3: 3.3% vs. 16.9% Gr3: 17% vs. 83%	>50%: 1.8% vs. 8.2%	NR	NR	NR	NR
2003; Tebeu [24]	NR	NR	NR	NR	NR	NR
2004; Tebeu [25]	NR	NR	NR	NR	NR	NR
2014; Shiozaki [26]	NR	<50%: 6.7% vs. 71.6% >50%: 3.3% vs. 18.1%	NR	NR	Yes: 0.3% vs. 0.3%	Yes: 7.9% vs. 41.8%
2022; Siegenthaler [27]	Gr1: 28.9% vs. 71.1% Gr2: 10.5% vs. 89.5% Gr3: 25.8% vs. 74.2% *	NR	NR	Positive: 42.4% vs. 57.6% *	Yes: 21.1% vs. 78.9%	NR
2023; Ueno [28]	NR	<50%: 53.7% vs. 67% >50%: 46.3% vs. 33% *	Absent: 76.6% vs. 81.9% Stromal: 23.4% vs. 18.01%	NR	NR	NR

NR: data were not reported in this study; * = statistically significant differences.

**Table 3:** Newcastle–Ottawa scale assessment.

Study	Selection				Comparability	Outcome		
	Representativeness of Cohort	Selection of Controls	Ascertainment of Exposure	Outcome Not Present at Onset	Comparability (Grade/DOI)	Assessment of Outcome	Adequate Follow-Up Period	Adequate Follow-Up
1983; Yazigi [16]	-	√	√	-	√√	√	√	√
1990; Grimshaw * [17]	-	-	-	-	--	-	√	√
1992; Kadar [36]	√	√	√	-	-√	√	√	√
1994; Ayhan [43]	√	√	√	-	--	√	-	√
1997; Descamps ** [19]	√	-	√	-	--	√	√	√
1997; Kashimura [18]	√	-	√	-	--	√	√	√
2001; Obermair [20]	√	√	√	-	√√	√	-	√
2001; Takeshima [9]	√	√	√	-	--	√	√	√
2003; Tebeu [24]	√	√	√	-	--	√	√	√
2003; Kasamatsu [7]	√	√	√	-	--	√	√	√
2004; Tebeu [25]	√	√	√	-	--	√	√	√
2006; Saga [21]	√	√	√	-	√√	√	√	√
2012; Gultekin ** [40]	√	-	-	-	--	√	√	√
2013; Garg [23]	√	√	√	-	--	√	√	√
2014; Shiozaki [26]	√	√	√	-	√√	√	√	√
2022; Siegenthaler [27]	√	√	√	√	--	√	√	√

* Congress abstract—no further data were available; ** peritoneal cytology was not the principal investigated factor; DOI: depth of myometrial invasion/cervical involvement.

## Data Availability

Data are available upon reasonable request.
